# Progress and Challenges of Point-of-Need Photonic Biosensors for the Diagnosis of COVID-19 Infections and Immunity

**DOI:** 10.3390/bios12090678

**Published:** 2022-08-24

**Authors:** Juanjuan Liu, Sebastian Wachsmann-Hogiu

**Affiliations:** Department of Bioengineering, McGill University, Montreal, QC H3A 0C3, Canada

**Keywords:** SARS-CoV-2, COVID-19, diagnostic, biosensors, photonics, point of need

## Abstract

The new coronavirus disease, COVID-19, caused by SARS-CoV-2, continues to affect the world and after more than two years of the pandemic, approximately half a billion people are reported to have been infected. Due to its high contagiousness, our life has changed dramatically, with consequences that remain to be seen. To prevent the transmission of the virus, it is crucial to diagnose COVID-19 accurately, such that the infected cases can be rapidly identified and managed. Currently, the gold standard of testing is polymerase chain reaction (PCR), which provides the highest accuracy. However, the reliance on centralized rapid testing modalities throughout the COVID-19 pandemic has made access to timely diagnosis inconsistent and inefficient. Recent advancements in photonic biosensors with respect to cost-effectiveness, analytical performance, and portability have shown the potential for such platforms to enable the delivery of preventative and diagnostic care beyond clinics and into point-of-need (PON) settings. Herein, we review photonic technologies that have become commercially relevant throughout the COVID-19 pandemic, as well as emerging research in the field of photonic biosensors, shedding light on prospective technologies for responding to future health outbreaks. Therefore, in this article, we provide a review of recent progress and challenges of photonic biosensors that are developed for the testing of COVID-19, consisting of their working fundamentals and implementation for COVID-19 testing in practice with emphasis on the challenges that are faced in different development stages towards commercialization. In addition, we also present the characteristics of a biosensor both from technical and clinical perspectives. We present an estimate of the impact of testing on disease burden (in terms of Disability-Adjusted Life Years (DALYs), Quality Adjusted Life Years (QALYs), and Quality-Adjusted Life Days (QALDs)) and how improvements in cost can lower the economic impact and lead to reduced or averted DALYs. While COVID19 is the main focus of these technologies, similar concepts and approaches can be used and developed for future outbreaks of other infectious diseases.

## 1. Introduction

***Impact of COVID-19 and importance of testing.*** If not diagnosed and treated properly, infectious diseases can become pandemic with significant implications to the economy and society. Rapid and accurate testing of infectious disease that are caused by pathogens is crucial for both patients and society, especially for infectious diseases that can spread easily. When infected individuals are identified quickly, patients can receive treatment immediately to avoid further deterioration, transmission is reduced, and adequate measures and restrictions can be implemented. COVID-19 is such an infectious disease that requires rapid and accurate testing. It has been, and it is still, affecting people’s lives worldwide since it was reported at the end of 2019 [1]. It is a respiratory disease caused by a novel coronavirus, Severe Acute Respiratory Syndrome CoronaVirus 2 (SARS-CoV-2). SARS-CoV-2 is highly contagious, thus leading to rapid transmission and increase in infected cases that burdens the health systems worldwide by the need for hospitalization and treatment. 

COVID-19 was declared a pandemic by the World Health Organization (WHO) in March 2020, and it has spread rapidly and affected most countries worldwide. To control the dramatic increase in infected cases, many governments have implemented various policies and restrictions such as lockdowns, social distancing, masks, and hand sanitizing. Inevitably, these measure affected people in many ways with regards to quality of life [2], mental health [3], economic situation, and other unintended consequences [4]. Up to date, more than 500 million COVID-19 cases have been reported globally, and more than 6 million people have died following infection with SARS-CoV-2. The direct impact of the disease burden of COVID-19 on the infected population can be measured using Disability-Adjusted Life Year (DALY). According to the published data from several countries (Scotland, Netherlands, Malta, Ireland, Germany, Denmark, and Australia), the DALY loss can reach up to 1980 DALYs per 100,000 people [5]. According to another study, the total DALY loss amount across 16 European countries reaches 4354 DALYs per 100,000 people, among which 98% is caused by the Years of Life Lost (YLL) [6]. Currently, approximately 12 billion vaccines have been administered worldwide. However, vaccinations alone are not sufficient to control the spread of COVID-19 since the virus is continuously evolving to form new variants that may affect its infectiousness. When the variants can increase transmissibility and/or decrease the effectiveness of current measures, vaccination, or therapeutics, etc., they are characterized as Variants of Interest (VOIs) and Variants of Concern (VOCs). According to the World Health Organization (WHO), currently circulating VOCs are subvariants of Omicron including BA.1, BA.2, BA.3, BA.4, and BA.5, after previously circulating VOCs of Alpha, Beta, Gamma, and Delta. Compared to previous variants, Omicron is more transmissive. Its subvariants BA.1, BA.2, and BA.3 have driven a fourth COVID-19 wave in South Africa, and now its new subvariants BA.4 and BA.5, especially BA.5, are driving a new wave globally [7]. 

To limit the increase in the cases and death, as well as to better protect the society against a continuously evolving SARS-CoV-2, the diagnosis of COVID-19 at the population level is important, with implications at both the individual and society levels.

**Figure 1:**
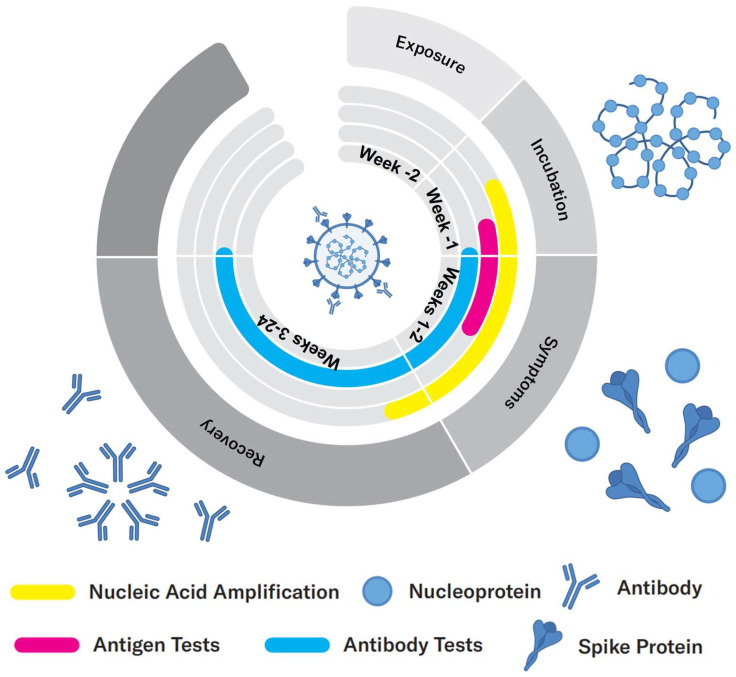
Schematic of the evolution of SARS-CoV-2 infection and timeline for detection via different types of tests. Taking the onset of symptoms as a baseline, it takes approximately 1–14 days from exposure with the virus to exhibit symptoms (exposure and incubation period in week -1 and week -2). The symptoms usually last for 1–2 weeks (weeks 1–2) for mild cases before recovery. For a severe case, the recovery may take a longer time, up to 6 weeks or more. The viral load starts to increase from incubation and peaks after the onset of symptoms; thus, the virus can be detected via nucleic acid tests and antigen tests during these periods. Antibodies are developed when the viral load is high and will last from weeks to months. Hence, the immune response can be detected via serological tests by detecting the developed antibodies (e.g., IgG and IgM).

***Evolution of the viral infection:*** According to Health Canada, it takes an incubation period of 1–14 days after exposure with the virus for a person to exhibit symptoms, during which time the viral load increases (Figure 1). During this period, the virus can be detected either by nucleic acid or antigen tests that identify viral components. Once the viral load is high in the body, antibodies are developed by the immune system, usually within a few days of infection, which allows for a serological test to be conducted, where the magnitude of the immune response is measured [8,9]. 

Therefore, since the outbreak of COVID-19, many researchers have adapted, improved, and developed new techniques and methodologies for the detection of COVID-19, either by viral tests, i.e., by detecting SARS-CoV-2 directly, or by serological tests, i.e., by detecting antibodies such as IgG and IgM [10]. Considering the rapid increase in infections worldwide, it is critical and urgent to develop biosensors for fast detection of SARS-CoV-2 at the point of need. Numerous researchers have devoted significant efforts to the development of various techniques to help facilitate the detection. There are three main types of testing methods for COVID-19 based on detecting specific viral nucleic acids, an antigen test that detects specific viral proteins, and a serological test that measures the presence of antibodies. Moreover, new testing methods that measure volatile compounds in the breath have also been developed and explored for COVID-19 diagnosis.

***Available testing methodologies:*** Currently, the most common methods are based on the detection of viral nucleic acids or proteins whereas samples are collected from a throat or nasal swab. ***Nucleic acid*** methodologies are the most accurate testing methods for the identification of SARS-CoV-2. The target analytes are viral RNA including different gene targets, such as ORF1 (opening reading frames) a/b and genes that are related to structural proteins (e.g., Spike protein, Nucleocapsid protein). To perform a nucleic acid test, nucleic acid amplification is needed to increase the detectable concentration after RNA extraction. A typical example of a nucleic acid test is Reverse Transcriptase Polymerase Reaction Chain (RT-PCR) [11], which is the gold standard for the diagnosis of COVID-19. In addition to thermal cycling, there are also other types of nucleic acid testing methods relying on isothermal amplification, such as LAMP (loop-mediated isothermal amplification), which is conducted at a constant temperature [12]. Further, the specific gene sequence will be detected via fluorescent labels. As a ‘gold standard’ method, nucleic acid detection provides a direct method for the diagnosis of COVID-19 by demonstrating the presence or absence of the viral genomes. It is the most commonly used technique for the diagnosis of COVID-19 with the highest sensitivity and specificity. Moreover, nucleic-acid-based diagnostic methods can provide reliable detection during the incubation phase of the virus, within a few days after the actual infection [13], which is earlier than other testing methods such as antigen and antibody tests that take longer to develop detectable analytes. Given the fact that COVID-19 is highly contagious, it is important to act as soon as possible once infection occurs to avoid further contact of the host with others. However, the drawback of this method is also obvious, which is that it is time- and labor-consuming. It takes typically more than 2 h to receive the results, and professional technicians are needed to perform the test with specialized lab equipment, leading to the need of significant human resources. Therefore, developing rapid nucleic acid tests or alternative rapid tests are important. ***Antigen tests*** provide an alternative tool for the rapid diagnosis of COVID-19. To perform an antigen test, the nasopharyngeal region is swabbed and antigen proteins specific to the virus are collected and detected. The biomarkers used for antigen tests are mainly the structural proteins on the surface of virus, among which the spike (S) protein and nucleocapsid (N) protein are most commonly targeted [14]. Compared to the nucleic acid test, the antigen test is less sensitive but provides simple and rapid detection, typically within 1 h. It is also easier to perform and does not require professional equipment, allowing for detection outside the laboratories at the point of need. Therefore, the rapid antigen test can satisfy the overwhelming demands of large-scale detection from all over the world. On the other side, it has lower accuracy, leading to lower confidence and more limited overall value. ***Antibody (serological) tests***, on the other hand, identify past, recent, or current infection with SARS-CoV-2 by indirectly detecting the antibodies produced by immune system against the virus, such as IgM and IgG. These antibodies are usually developed within a few days after the infection with the virus. More specifically, it takes typically 5–7 days to develop IgM antibodies and a longer time (10 days or longer) for IgG antibodies. IgM and IgG antibodies can then persist for weeks and months, respectively [8,9]. In other words, these antibodies can remain in bloodstream and be detectable after the infected patients have recovered. As a result, although its detection mechanism is similar to the antigen test, i.e., via a biorecognition event, it cannot be used as a similar diagnostic method to the antigen test. Instead, antibody tests can identify previous infections by evaluating the immune response. Even though the response of the immune system is complex and not fully understood, these tests are still important for research, and for the overall monitoring of the infected cases. ***Other testing methodologies****:* Infection with the virus may cause, in certain persons, metabolic changes that, while non-specific to the particular virus, can be utilized to indirectly and qualitatively detect the infection [15]. Such tests that measure metabolites or their impact on the volatile compounds in breath hold a particular appeal due to the potential for non-invasive and rapid detection. As a recent example, a test that measures and analyses the spectra of organic volatile compounds in breath has been reported for COVID-19 detection [16]. Compared to the abovementioned testing methods, new techniques providing an alternative window of COVID diagnosis and screening by utilizing different approaches have also been proposed, such as a breathalyzer. A breathalyzer detects the presence of volatile organic compounds (VOCs) in the ketone and aldehyde families that are associated with SARS-CoV-2 infection. Recently, the FDA has approved the first breathalyzer, known as the InspectIR COVID-19 Breathalyzer. 

***Scope of the article:*** The development of biosensors can facilitate the diagnosis of COVID-19 via different analytes. Considering the high contagiousness of SARS-CoV-2 and massive testing requirements, it is important to develop detection techniques that are accurate and rapid. In addition, to perform detection at the point of need is also significant so that the test can be performed in various environments such as airports, public events, or even at home, to prevent the spread of disease. As a result, cost-effectiveness plays an important role in the development of such techniques. Based on the transducer, there are different types of biosensors such as piezoelectric [17], electrochemical [18], and optical biosensors [19]. Photonic/optical biosensors are advantageous for their high sensitivity, specificity, and rapid response. Numerous researchers have designed and developed photonic biosensors for the diagnosis of COVID-19. Therefore, in this review article, we present photonic techniques that have been used or are currently explored for COVID-19 diagnosis, describing their main fundamental principles of operation and performance for specific use cases. With many biosensors having been approved for emergency use, and significant research and funding allocated for the development of clinical SARS-CoV-2 biosensors, we further discuss the clinical characteristics and readiness level of photonic techniques for COVID-19 diagnosis. This will provide the reader a profound insight into the advantages, limitations, and challenges for the development of photonic biosensors that are aimed for commercialization and/or implementation in practical applications. 

## 2. Characteristics of a Biosensor

A biosensor is a device that uses a biorecognition element, a transducer, and a detection system to detect and quantify an analyte of interest. The biorecognition element can be antibodies, nucleic acid sequences, or even whole cells. The transducer, on the other hand, utilizes a physical, chemical, thermal, or electrical measurement to detect a change in these parameters upon a binding event of the analyte of interest to the bioreceptor. 

An analytical biosensor is defined by several parameters or characteristics that indicate its performance and, in the end, its accuracy for a specific application [20]. These parameters are then used by regulatory bodies to determine whether the specific requirements are met for approval. Based on the development stage, there are technical and clinical characteristic parameters that are used for design in the research stage and clinical applications, respectively. Therefore, in this section, we will discuss technical and clinical characteristic parameters separately.

### 2.1. Technical Characteristics of a Biosensor

Technical characteristics are the parameters that are closely related to the sensing performance and used to evaluate a biosensor for different use cases. 

*Sensitivity:* sensitivity is a significant parameter that characterizes a biosensor. It describes how a biosensor responds to the presence of a target analyte. It is the relationship between the detected signal and the concentration of analyte. More specifically, it is the ratio of the corresponding change in detected signal (such as light intensity in an optical biosensor) to the change in analyte concentration. For the linear detection range, the sensitivity is the slope in a calibration plot that is obtained by performing experiments at a series of concentrations of the analyte. With higher sensitivity, a biosensor can better detect the change in concentration, i.e., it provides better quantification with higher precision and accuracy. The sensitivity is determined by many parameters such as: the ability of the transducer to detect very small physical or chemical changes, the dynamic range of the transducer, the affinity of the biorecognition element towards the target analyte, and the enhancement factor of a substrate.

*Limit of detection (LOD):* LOD refers to the lowest quantity of the target analyte that can be detected by a biosensor. More specifically, it is the lowest concentration of an analyte that can cause a measurable signal by the transducer where this signal is extractable and distinguishable from the signal measured in the absence of the analyte, i.e., the blank experiment. Different from limit of quantification (LOQ), which is the lowest concentration that can be distinguished with acceptable accuracy and precision, LOD indicates a measurable signal, but not necessarily one quantified with acceptable accuracy. For the detection of analytes within a linear concentration range, the value of LOD is calculated as 3 SD/ S (as a comparison, LOQ = 10 SD/S), where SD is the standard deviation of a blank, and S denotes the slope of the calibration plot. LOD plays an important role for a biosensor, especially in medical applications where a low concentration of biomarkers needs to be detected. LOD is determined mainly by the transducer technology and the specificity of interaction between the analyte and bioreceptor. *Linear detection range:* linear detection range, or linearity, not only describes the applicable concentration range of an analyte that a biosensor can detect, but also determines the accuracy of the measurements that is related to the slope of the calibration plot. The linear detection range can be obtained by measuring different concentrations at a wide range, and then performing a linear fit with an acceptable R square (coefficient of determination). The linear detection range is useful to determine potential applications of the biosensor. For example, to detect urea in medical diagnosis, the linear detection range of a biosensor needs to include the physiological range of urea.

*Specificity and selectivity:* specificity refers to the ability of a biosensor to respond specifically to the target analyte. It can be achieved with bioreceptors that have high affinity for the target analyte, for example, specific antibody–antigen or aptamer–antigen interactions, or the utilization of enzymes that catalyze specific reactions. Selectivity, on the other hand, is the ability to detect the analyte in the presence of other interference substances. It is often characterized by comparing the detected signals of an analyte in the absence and presence of other interference components. 

*Reproducibly:* reproducibility represents the consistency of measurements that are performed when using the same methodology and conditions. It indicates the ability of a biosensor to obtain similar results when repeating the experiment. Reproducibility can be calculated using the Relative Standard Deviation (RSD) or Coefficient of Variance (CV) of a group of measured results obtained from repeated experiments. Based on the selected group of results, reproducibility can evaluate the variation of measurements from sample to sample, time to time, substrate to substrate, or spot to spot. The reproducibility can be affected by non-uniformities in the substrate and/or the manufacturing error of substrates.

In addition to the parameters mentioned above, other characteristics such as cost, response time, and ease of use are also important for the application of a biosensor, especially when considering its application in practice. For example, for COVID-19, a biosensor at the Point-Of-Need (PON) will significantly benefit its testing at massive scale. As a result, to design such a biosensor, low cost, rapid response, and simple operation will be considered.

### 2.2. Clinical Characteristics of a Biosensor

The technical characteristics described above are important parameters for the design of a biosensor in a research lab. However, for a biosensor that is designed for practical application to medical diagnosis, more parameters need to be evaluated and validated before implementation for clinical applications. In a binary (yes/no, or infected/healthy) situation, their testing performance can be characterized by clinical sensitivity and specificity, which are the True Positive Rate (TPR) and True Negative Rate (TNR) when a gold standard exists, or Positive Percent Agreement (PPA) and Negative Percent Agreement (NPA) when no reference standard exists for comparison. Sensitivity describes the ability of the device or a biosensor to identify the actual infected people, whereas specificity describes its ability to identify the healthy people. In addition, the performance of the devices or biosensors can also be characterized by Positive and Negative Predictive Values (PPV and NPV). PPV, also known as precision, is defined as a ratio of the true positive results to all the positive results (including both true positive and false positive) and it demonstrates the probability that an individual testing positive is indeed infected, whereas NPV is the ratio of the true negative results to all the negative results, and it indicates the probability that an individual who tests negative is not infected. The definition of these parameters is listed as below: 

In Table 1, the cell highlighted in grey with 2 × 2 columns and rows is also known as a confusion matrix, which presents the classification accuracy of a biosensor or device by visualizing the actual and test results (infected or healthy). In addition to sensitivity and specificity, there are other parameters that are used for the performance evaluation of a clinical biosensor, such as False Omission Rate (FOR, = $\frac{FN}{TN+FN}=$ 1 − NPV), False Discovery Rate (FDR, = $\frac{FP}{TP+FP}$ = 1 − PPV), accuracy (ACC, = $\frac{TP+TN}{Total\;population}$ ), or prevalence (=$\frac{TP+FN}{Total\;population}$). Among these parameters, accuracy and prevalence are particularly important for the clinical biosensor or device used in a pandemic, since accuracy describes how close a measurement is to the true value and prevalence predicts the proportion of infection among the detected population.

In addition, it is also important to evaluate the binary classification performance of a medical device to evaluate its reliability to be used for decision making. A receiver operating characteristic (ROC) curve is a plot that helps evaluate whether a case can be considered infected or not infected by identifying the optimal cut-off threshold for classification. It is defined as a plot of TPR (sensitivity) versus False Positive Rate (FPR, 1- specificity) at different thresholds. The Area under the Curve (AUC) of the ROC indicates the probability of correct classification of a random sample. Hence, it shows overall the classification performance at different thresholds of a binary classification system. 

## 3. Fundamentals of Photonic Biosensors

Photonic sensors employ transducers that use optical phenomena to detect the analyte of interest by measuring the change in optical properties, based on which optical sensors can be classified into different types such as luminescence (measuring the emission of light), colorimetric (measuring the change in absorption of color), refractometers (measuring refractive index changes—interferometers are an example), spectroscopic (measuring spectral changes), and other types of technologies. In this section, we summarize the most relevant optical methods including plasmonic, luminescence/fluorescence, colorimetric, imaging/microscopy, interferometric, ring resonator, and photonic crystals-based techniques. Here, we review the detection methods that have been reported for COVID-19 diagnosis (Table 2), with emphasis on the working principles of the detection and exploration for the diagnosis of COVID-19 in the lab. Moreover, we discuss how each photonic technique has been developed and implemented for different testing methods with various analytes.

### 3.1. Luminescence: Fluorescence- and Chemiluminescence-Based Biosensors

**Figure 2:**
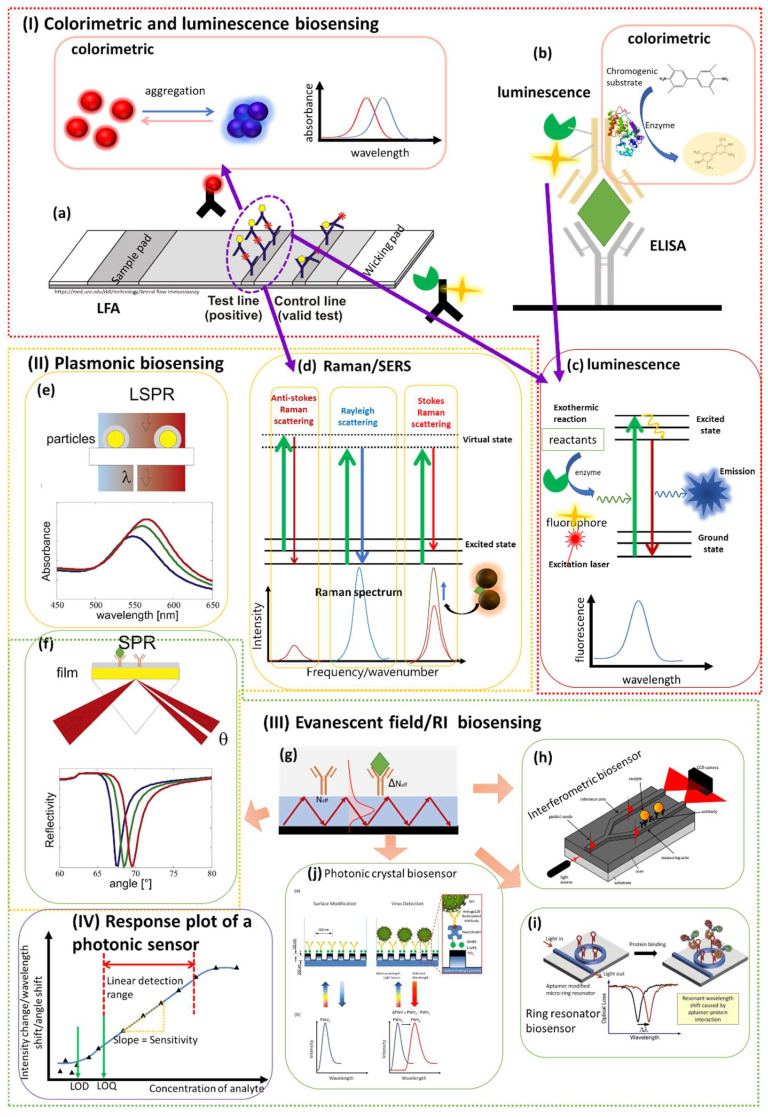
**Photonic biosensors based on different techniques**. (**I**) colorimetric and luminescence biosensors: (**a**) working principle of colorimetric biosensor based on aggregation on a LFA platform; (**b**) working principle of colorimetric biosensor based on chromogenic substrate in ELISA; (**c**) basic principle of luminescence process driven by chemical reaction (chemiluminescence, left top) and photon excitation (fluorescence, left bottom). (**II**) Plasmonic biosensors: (**d**) basic principle of Raman scattering and SERS; (**e**) working principle of LSPR; (**f**) working principle of SPR. (**III**) evanescent field/ refractive index-based biosensors: (**g**) mechanism of evanescent filed sensing; schematics of working principle of (**h**) interferometric biosensor, (**i**) ring resonator biosensor, and (**j**) photonic crystal-based biosensor. (**e**,**f**) are adapted from ref. [103] with permission from Elsevier, Copyright (2016). (**h**) is adapted from ref. [104] with permission from Universiteit Twente. (**i**) is adapted from ref. [105] with permission from Elsevier, Copyright (2012). (**j**) is adapted from ref. [106] with permission from Springer Nature, Scientific Reports, Copyright (2014). (**IV**) A typical calibration plot of a photonic biosensor.

Luminescence is a phenomenon where light is emitted by a molecule when it returns to the ground state after being excited, along with the release of energy in the form of photon emission [34] (Figure 2c). Based on the mechanism by which the molecule is excited, there are different types of luminescence. One of the widely known phenomena is chemiluminescence, where the excitation is achieved via a chemical reaction, also known as bioluminescence when taking place in a living organism. In a chemiluminescence reaction, a reactant is oxidized with the generation of a highly reactive product or intermediate that is excited. The product or intermediate in an excited state then quickly decays to the ground state by producing light, which is observed as light emission [35]. To fulfill the requirement for a chemiluminescence reaction for light emission, the chemical reaction must be exothermic and release sufficient energy to excite the intermediate molecule, and this excited molecule will emit photons while returning back to the ground state [36].

It is a complex process involving multiple steps. Taking luminol as an example, it exists as an anion with two negative charges/electrons that are delocalized on oxygen in the enol-form. When the enol-form luminol anion is oxidized by molecular oxygen, an unstable cyclic peroxide is produced and quickly decomposes to nitrogen and 3-aminophthalic acid with electrons in an excited state. When the excited state relaxes to the ground state, the excess energy is released as a photon with a wavelength of approximately 425 nm wavelength. 

In the case of bioluminescence, which is a special type of chemiluminescence that produces light in living organisms, an enzyme known as luciferase is utilized to catalyze the reaction of luciferin and other co-factors to produce light emission. Depending on the different species of the organisms, the conditions needed for the bioluminescence reaction can be different. For example, the firefly luciferase reaction is one of the most well-known bioluminescence reactions, and it relies on ATP (adenosine triphosphate) that is generated by living organisms and magnesium ions as well for the reaction. This bioluminescence reaction is a two-step process, where d-luciferin is firstly bound to luciferase and undergoes adenylation (luciferyl-AMP) [37]. The adenylation of bound d-luciferin leads to the formation of carbanion on the thiazoline ring [38]. It is then reacted with molecular oxygen and then produces a cyclic peroxide. When the cyclic structure breaks, oxyluciferin in an excited state is produced in either enol or keto form with the release of CO_2_. When the excited states return to the ground state, yellow–green light and red light are emitted by the enol and keto form, respectively [39]. However, for the Renilla luciferase reaction system, which is used in sea pansy, only the luciferin substrate, i.e., coelenterazine, is required to start the bioluminescence reaction.

Chemiluminescence biosensors utilize these reactions, such as the luminol–hydrogen peroxide system and luciferin–luciferase system, to produce luminescence as the detection signal. To detect the presence and concentration of the target analyte via chemiluminescence intensity, it is necessary to establish the relation between the analyte and the reaction system. This can be achieved mainly in two ways. The first one is where the analyte or the product of the analyte is involved with the reaction as a reactant, enzyme, or other cofactor for the reaction to occur. For example, the oxidation of glucose can produce hydrogen peroxide, which can be used to oxidize luminol for chemiluminescence [40]. In another way, the analyte is not participating in the reaction, but labeled with the reagents such as enzymes that are involved with the reaction to produce emission. For example, by labeling the enzyme horseradish peroxidase (HRP) with a capture antibody, the cancer antigen can be detected via a chemiluminescence reaction that is catalyzed by HRP upon antibody–antigen binding [41]. Once the chemiluminescence reaction occurs, the emitted photons are collected and analyzed using a photodetector such as a CCD, CMOS sensor, or photomultiplier tube (PMT). 

Another well-known type of luminescence is photoluminescence that absorbs photons to excite emission. There are two types of luminescence, fluorescence and phosphorescence. Fluorescence is more commonly used for the development of a biosensor. In fluorescence, the excitation of molecules is achieved by incident photons with a wavelength (excitation wavelength) matching the absorption spectrum. The molecule is excited to a higher energy level after absorbing the incident light, and then returns to the ground energy state with light emission typically at a longer wavelength (emission wavelength). Considering the fact that most intrinsic fluorescence from the analyte is weak, fluorescence biosensors normally utilize fluorescent molecules (fluorophores) as a tag for the analyte for detection [42]. 

For luminescence-based biosensors, the detected intensity of photon emission is determined by the generation efficiency of the excited molecules that can emit light. In the case of fluorescence, it is dependent on the quantum yield *Φ_f_* = emitted photon number/absorbed photon number, and the fluorescence intensity is calculated as following [43]:

*I_f_ = Φ_f_ (I_i_* − *I_t_)* (1)

where *I_i_* and *I_t_* represent the intensity of incident light and transmitted light, respectively. According to Beer–Lambert law [44], *I_t_* = 10*^−εlc^ I*_i_, where *ε* is the molar absorption coefficient, *l* is the optical path length, and *c* is the concentration of the analyte in solution. Therefore, the fluorescence intensity can be further expressed as following:

*I_f_ = Φ_f_ I_i_ (1* − *10^−εlc^)* (2)

In the case of chemiluminescence, it is more complex since more steps are involved during the chemical reaction. The photon emission intensity can be expressed in the following equations [36]:

(3) $$I_{CL}=\Phi_{CL}-\frac{dA}{dt}$$

*Φ_CL_ = Φ_R_ × Φ_E_ × Φ_f_* (4)

where *I_CL_* is the emission intensity, *Φ_CL_* is chemiluminescence quantum yield, $\frac{dA}{dt}$ is the consuming rate of the precursor (such as luminol), *Φ_R_* = number of reacted molecules undergoing the chemiluminescent pathway/total number of all reacted molecules, *Φ_E_* = number of molecules forming electronically excited product/number of reacted molecules undergoing the chemiluminescent pathway, *Φ_f_* is the quantum yield of the light-emitting species that is equal to the ratio of emitted photons to the absorbed photon. This definition of the chemiluminescence quantum yield is for the direct chemiluminescence reaction where the produced product is excited. In the case of indirect reactions, where light is produced by a secondary excited product from the direct excited product, the energy transfer rate should also be considered.

As shown above, the luminescence intensity is a function of the chemical/fluorophore concentration. Therefore, luminescence-based biosensors can be used for quantitative detection. There are many luminescence-based biosensors developed for serological tests. Chemiluminescence immunoassay is a typical example that is commonly explored [45]. It has already been studied for commercial use such as in the Abbott AdviseDx and Roche Elecsys SARS-CoV-2 tests [21]. The Abbott AdviseDx SARS-CoV-2 IgG II assay can be used to help identify recent or prior infection with SARS-CoV-2 by detecting IgG antibodies in serum or plasma. The target IgG antibody will bond to anti-human IgG acridinium-labeled conjugate, which is the chemiluminescent molecule for detection. However, it can be used under emergency by laboratory professional only and the identification is semi-quantitative. Thus, it is not applicable for clinical use yet. The Roche Elecsys SARS-CoV-2 test is an electrochemiluminescence assay that utilizes a ruthenium complex (Tris(2,2′-bipyridyl)ruthenium(II)-complex (Ru(bpy))) as an electrochemiluminescent probe. It forms a sandwich structure with the target antibodies to SARS-CoV-2 and biotinylated SARS-CoV-2 specific recombinant antigen, which will be later bound to streptavidin-labeled magnetic microparticles. However, these assays still require specialized instruments for further analysis.

In addition to chemiluminescence-based biosensors, fluorescence-based biosensors are also widely explored. For example, Wang et al., proposed a fluorescence-based lateral flow immunoassay for simultaneous detection of SARS-CoV-2 S and N protein antigens utilizing a spike-protein-conjugated quantum dot as a fluorescence nanotag [46]. The fluorescence nanotag is composed of a silica core and double quantum dot shell layers. The S-protein-conjugated nanotag will form a sandwich complex with SARS-CoV-2 IgG/IgM and the immobilized anti-SARS-CoV-2 IgG/IgM on the test lines in the presence of SARS-CoV-2 IgG/IgM antibodies. The results are then collected using a fluorescence reader with excitation lasers. Further, they reported another fluorescence lateral flow immunoassay using a magnetic quantum dot with a triple quantum dot shell (MagTQD) as a nanotag for the simultaneous detection of SARS-CoV-2 S and N protein antigens [23]. It allows for dual-mode detection, i.e., a direct detection mode and a magnetic enrichment detection mode, to fulfill the requirements for different applications and purposes. The detection limit is demonstrated to reach the pg/mL level. 

### 3.2. Colorimetric Biosensors

A colorimetric biosensor measures the change in color of the indicator or sensing probe upon binding of the analyte to the bioreceptor [47]. The colorimetric indicators can be any materials or substances that exhibit certain colors or can produce color change within visible range, such as chemical dyes, plasmonic nanoparticles (e.g., AuNPs or AgNPs), quantum dots, and polymers [48,49,50,51]. 

Quantitative detection can be achieved by directly measuring the intensity change of the indicators that are conjugated with target analytes, or by a colorimetric transition that is induced by the interaction of indicators with target analytes. 

Plasmonic nanoparticles are commonly used as a reporter in colorimetric biosensors due to the color change that is related to their optical properties. For example, the color of gold nanoparticles is determined by their size, shape, and surface chemistry. The incident light interacts with the free electrons in the nanoparticles and induces localized surface plasmon resonance that absorbs light at resonance wavelengths. For the nanoparticles with smaller size, the resonance wavelengths are shorter. Thus, the light at shorter wavelengths is absorbed due to resonance and a red color is shown. By contrast, for large-sized nanoparticles, the color changes towards purple and blue. Therefore, a nanoparticle-based colorimetric biosensor can be designed depending on the size or morphology changes such as chemical etching or growth that are induced or mediated by the target analyte [52,53,54,55]. In addition, the distribution of nanoparticles also affects the optical properties of the nanoparticle. In detail, the aggregation of nanoparticles will lead to dipole–dipole interaction and coupling of plasmons generated in the neighboring nanoparticles, thus affecting the absorbance and leading to redshift [56] (Figure 2a). Colorimetric biosensors based on an aggregation strategy are commonly explored [55], where the target analyte induces aggregation based on crosslinking by binding to functionalized nanoparticles [57], or based on a non-crosslinking mechanism by directly acting as a stabilizer [58]. In addition to gold nanoparticles based on surface plasmon resonance, there is another type of color change related to optical properties by utilizing thin film interference color change. When interference occurs between the reflected light on the upper and bottom interface of a thin film substrate, the interference color is determined by the thickness and refractive index. Therefore, by controlling the thickness via the surface binding activities of analytes, the color change can be achieved and used for detection [59]. 

Moreover, colorimetric biosensors can also be developed based on enzyme probes that catalyze the enzymatic reaction of a chromogenic substrate to generate a color product for detection [60] (Figure 2b). Commonly used chromogenic substrates with HRP are TMB (3,3′,5,5′-tetramethylbenzidine), 4-CN (4-chloro-1-naphthol), and DAB (3,3′-diaminobenzidine tetrahydrochloride) [61]. There are also other chromogenic substrates used with another enzyme, alkaline phosphatase (AP). These chromogenic substrates are oxidized in a colorimetric reaction in the presence of enzyme to produce a colored precipitate. For example, in the presence of HRP, colorless TMB can react with hydrogen peroxide and is oxidized to generate an intermediate (charge-transfer complex) exhibiting blue color with a maximum absorbance at 652 nm. It also generates another colorless intermediate that is further oxidized into a diamine oxidation product exhibiting yellow with a maximum absorbance at 450 nm. The color change is captured by imaging and its intensity is further analyzed for quantification. The color change usually can be monitored by visual examination and can be further quantified by a spectrometer such as a UV-Vis spectrometer that measures the change in absorption spectra.

Compared to other types of biosensors, colorimetric biosensors offer a straightforward method for analyte detection that can be easily identified visually. Therefore, the development of colorimetric biosensors attracts a lot of attention from both researchers and the market. It also attracts attention for the diagnosis of COVID-19, since it provides rapid detection at the point of need that is beneficial for preventing the transmission of COVID-19. The development of colorimetric biosensors aims for the detection of surface proteins such as spike protein as well as the antibodies against SARS-CoV-2. Ventura et al., proposed a colorimetric biosensor to detect spike, envelope, and membrane proteins on the surface of the virus. This biosensor is based on the aggregation of gold nanoparticles that are functionalized with a polyclonal antibody against the target proteins [62]. 

### 3.3. LFA and ELISA

Lateral Flow Assay (LFA) has been explored as a common commercial product. LFA is developed on a cellulose-based matrix that is typically composed of several sections: the sample pad, the conjugate pad, the test line, the control line, and the absorption/wicking pad [63]. The detection mechanism of LFA is based on a probe that is conjugated with colored reporter such as gold nanoparticles (AuNPs) and latex beads [64,65]. By detecting the color intensity of the probe, the concentration of the analyte can be measured. To label the probe on the analytes and further build a relationship between the concentration of the analyte and the reporter, two formats of assays can be developed, establishing a direct or competitive relationship, respectively. For the direct detection of analyte, a sandwiched assay is formed by two complementary antibodies against the target analyte. One of the complementary antibodies is known as the detection antibody, and it is conjugated with the colored reporter to act as a probe. The detection antibodies are usually distributed on the conjugate pad. When the sample containing analytes flow through the conjugate pad from the sample pad, the detection antibodies with reporter will be bound to the analytes and flow towards the test line. The second antibody is immobilized on the test line to capture analytes. Therefore, it is also called the capture antibody. When the samples reach the test line, only the complex of reporter–detection antibody–analyte will be captured, and the other antibodies without analyte will not be detected here. The test line will exhibit color from the reporter particles that are labeled on the analyte [66]. By analyzing the intensity of the test line, the concentration of analytes can be obtained. However, when small molecules are detected, it is challenging to bind them to two antibodies. To address this issue, a competitive LFA can be used. In the case of a competitive assay, the immobilized antibodies on the test lines can be considered as available spots while the analyte and the conjugated antibody with a reporter will compete for the immobilized antibodies on the test line [67]. Therefore, the detected color intensity is inversely proportional to the concentration of analytes. 

Enzyme-linked immunosorbent assay (ELISA) is an analytical technique that detects the analyte (ligand) via an enzymatic reaction. The measured signal for ELISA comes from the reaction of a chromogenic substrate such as tetramethylbenzidine (TMB) that needs to be catalyzed by an enzyme (e.g., horse radish peroxidase (HRP)) [68]. The enzyme is labeled on a detection antibody that can be specifically bound to the target analyte in a direct or indirect way. Depending on how the target ligand is linked with the enzyme, there are four different types of ELISA tests. The simplest one is a direct ELISA test where the analyte is adsorbed on the surface of the plate and then bound to the detection antibody with the labeled enzyme. By detecting the color intensity produced in the enzymatic reaction that is proportional to the captured enzyme by the analyte, the concentration of the analyte can be assessed. The second type is an indirect ELISA test. Instead of direct binding of analyte to the enzyme-labeled antibody, indirect ELISA uses a secondary antibody to be labeled with the enzyme. The labeled secondary antibody is then bound to the primary antibody that is recognized by the target ligand. Another type of ELISA is more similar to the construction structure used in LFA, where a sandwich assay is developed. A capture antibody is immobilized on the plate to capture the analyte, and a detection antibody with a labeled enzyme is used for measurements. During measurement, the analyte will be sandwiched in between two antibodies [69]. Compared to the previous tests, the sandwich format improves the specificity of analyte detection in the presence of other substances. Lastly, there is also a competitive ELISA test. For a competitive ELISA test, the capture antibody is still immobilized/coated on the plate, but the sample containing the target antigen will be added together with enzyme-conjugated antigen to the plate. The enzyme conjugate will compete with the target antigen for the binding availability on the capture antibody. The detected signal will be directly related to the bound enzyme–antigen conjugate, and the binding of the target antigen will block the binding of the conjugate. Therefore, the signal intensity is reversely related to the concentration of the target analyte. 

It is also worth mentioning that, for both LFA and ELISA, colorimetric detection is one of the detection methods. They can also be combined with other detection techniques such as fluorescence and SERS to achieve better quantification. To use a different technique, the reporter molecule needs to be active towards the specific technique. For example, a fluorescent tag should be used for fluorescence detection.

Gold nanoparticles are traditional reporters and enhancers used for LFA detection [66,70,71,72]. To improve the specificity and sensitivity, gold nanoparticles are typically conjugated with the antibodies that are specific for the analytes of interest. Research has been reported using colloidal gold nanoparticles to construct an LFIA detection system for the IgM antibody against the SARS-CoV-2 virus [25]. To further improve sensitivity, research exploring other particles for the LFIA probe is also reported. Chen et al., proposed an LFIA system utilizing lanthanide-doped polystyrene nanoparticles to detect anti-SARV-CoV-2 IgG in human serum [26]. Wang et al., developed an LFIA system for IgG and IgM detection using selenium nanoparticles, which exhibit orange color that is visible to naked eyes [27]. However, quantitation via LFIA is problematic. Therefore, LFA with different reporter systems, such as fluorescent probes, have also been explored to address these issues [23]. Chen et al., developed a near-infrared emissive LFIA against SARS-CoV-2 based on aggregation-induced emission nanoparticles to detect IgG and IgM [73]. A CMOS detector is used to record the fluorescence signal on the test line. Bayin et al., on the other hand, built an LFIA platform for IgG and IgM detection with superparamagnetic nanoparticles (SMNPs) and a giant magnetoresistance (GMR) sensing system [28]. Both coloration and magnetic signals can be obtained. The GMR sensing system allows for quantitative measurements. 

### 3.4. Plasmonics: Raman/SERS, LSPR, and SPR

Plasmonic techniques utilize Surface Plasmons (SPs) that are generated at the dielectric–metal (such as air–metal) interface as the coherent oscillation of electrons in the metal induced by the oscillating electric field from the incident light [74]. Depending on the structure of the metallic materials at the interface, there are localized surface plasmons and propagating surface plasmons. Localized surface plasmons are surface plasmons generated and confined at the surface of metallic nanoparticles, while propagating surface plasmons are surface plasmons that propagate along the surface of the metal, which is typically a thin film. Based on the utilized phenomena, there are different types of plasmonic techniques, further leading to different types of biosensors. The commonly known methods such as the Surface-Plasmon-Resonance (SPR)- and Localized-Surface-Plasmon-Resonance (LSRP)-based techniques show high sensitivity and have attracted a lot of attention for viral detection [75]. In addition, Raman-spectroscopy-based technologies such as Surface Enhanced Raman Spectroscopy (SERS) are also widely used and studied for viral detection due to their high specificity and sensitivity [76]. 

*SPR:* A Surface-Plasmon-Resonance (SPR)-based biosensor utilizes a propagating surface plasmon polariton (SPP), which refers to the electromagnetic field that is coupled with the surface plasmon. In the Kretschmann configuration that is commonly used for SPR detection, it is composed of several layers including a prism where the light is incident, a metal layer on a glass support, and the sample on the surface of the metal, i.e., at the interface of metal and dielectric (air). When the light of a certain wavelength (*λ*) is incident at the interface dielectric and planar metal at a certain angle (*θ*), the wavevector of the evanescent wave ($k_{evan}$ = $\frac{2\pi }{\lambda }n_{p}\sin\left(\theta \right)$) generated upon the incidence can be calculated as a function of the incident angle, wavelength, and the refractive index of the prism ($n_{p}$) [77]. On the other hand, the wavevector of the surface plasma wave ($k_{SP}=\frac{\omega }{c}\sqrt{\frac{n_{D}^{2}n_{M}^{2}}{n_{D}^{2}+n_{M}^{2}}}$, where ω is the angular frequency of the wave and c is the speed of light in a vacuum) generated by the propagating surface plasmon at the metal–dielectric interface can be described as a function of the refractive indexes of the dielectric ($n_{D}$) and metal ($n_{M}$) [78]. When total internal reflection occurs, the incident electromagnetic wave is in resonance with the coherent electrons. As a result, $k_{evan}=k_{SP}$, and the incident angle *θ* can be calculated to be related with the refractive indexes of the metal and dielectric at the interface: $\theta_{SPR}=\arcsin\left(\frac{1}{n_{p}}\sqrt{\frac{n_{D}^{2}n_{M}^{2}}{n_{D}^{2}+n_{M}^{2}}}\right)$ [42]. Therefore, the change in the refractive index at the interface will cause the change in this angle (SPR angle or resonance angle, as shown in Figure 2f). The SPR-based technique thus utilizes this feature in biosensing. Specifically, when a binding event or adsorption of analytes occurs at the interface, the refractive index is changed. The change can be reflected in the change in the resonance angle. In an SPR biosensor, the angle shift is used to measure the analyte regarding the binding event, adsorption, and concentration. Thus, quantitative measurements can be achieved by a SPR biosensor. 

Since SPR measures molecular interactions such as antibody–antigen reactions, it is a suitable tool for the detection of associated antibodies against SARS-CoV-2 or the detection of surface proteins of SARS-CoV-2. So far, theoretical and experimental studies on SPR biosensors established using different structures and materials are reported. However, the progress of SPR biosensors developed for COVID-19 is still in early stage. Most studies are focused on theoretical modeling and numerical analysis.

As an example, a theoretical analysis of SPR has been reported for the design of SPR biosensors with improved sensitivity. With a basic Kretschmann layout, Das et al., reported the investigation of SARS-CoV-2 utilizing Au nanorods (AuNRs) that are conjugated with the SARS-CoV-2 spike protein antibody on an Au-nanosheet-coated prism to amplify the detected signals via SPR immunosensor [29]. The SARS-CoV-2 virus is captured and sandwiched in between the immobilized spike protein antibody and AuNR-conjugated antibody. Uddin et al., designed a modified Kretschmann configuration for an SPR biosensor that combines layers of BaTiO3 and silicon on the Ag-coated BK7 prism, and further evaluated its sensing performance by numerical analysis [79]. The bio-recognition element to identify SARS-CoV-2 is a thiol-tethered DNA ligand that is functionalized on the surface of the top BaTiO3 layer. Similarly, Akib et al., reported a theoretical model of SPR biosensors using a modified Kretschmann configuration that can be used for the detection of COVID-19. This biosensor is developed by incorporating layers of graphene and platinum-di-selenide (PtSe2) on the top of an Au-coated prism and it has been designed to be versatile for COVID-19 detection using different types of analytes and receptors such as virus spike proteins with IgG [80].

In addition to the theoretical modeling and evaluation of the design of SPR biosensors for COVID-19 detection, efforts have also been devoted to identifying potential improvements of SPR biosensors for COVID-19 detection. Djaileb et al., developed a SPR biosensor for the detection of IgG antibody in different samples including serum, plasma, and dried blood spots by measuring the interaction between SARS-CoV-2 proteins and the associated IgG antibodies [81].

*LSPR:* A Localized-Surface-Plasmon-Resonance (LSPR)—based biosensor utilizes localized surface plasmons that are generated at the surface of metallic nanoparticles. When the incident light waves at a certain wavelength are in resonance with induced oscillating electrons in metallic nanoparticles, the light will be absorbed, causing a decrease in the reflectivity at this wavelength. Similarly to SPR, the resonance condition is very sensitive to changes at the metal–dielectric interface. Upon the interaction of the analyte with bioreceptor molecules at the interface, the wavelength that fulfills the resonance condition changes, inducing a shift in wavelength or decreased reflectivity at a specific wavelength (Figure 2e). In an LSPR configuration, the incident light usually consists of a broad range of wavelengths such as white light. The reflected light is collected by an optical detector and the resonance wavelength is detected by measuring the decreased reflectivity.

There is also research reported on the development of LSPR-based biosensors for identification of COVID-19. Qiu et al., developed a dual-functional biosensor for the detection of a selected sequence from SARS-CoV-2 by combining the plasmonic photothermal heat effect for nucleic acid hybridization. Au nanoislands were used as the sensing substrate and were functionalized with thiol-cDNA that was complementary with the target sequence, the RdRp-COVID sequence. A differential phase response corresponding to the change in the refractive index at the interface of the Au nanoislands due to the binding event of target virus was recorded and analyzed [82]. It was demonstrated to be able to achieve a LOD as low as the pM range. Funari et al., reported an LSPR biosensor for the detection of antiviral antibody against the SARS-CoV-2 spike protein [30]. They electrodeposited Au nanospikes on a glass substrate and then integrated it with a microfluidic chip for sample injection and connection to light source and detector. 

*Raman/SERS:* Raman spectroscopy is an analytical technique that determines analytes based on an optical phenomenon known as Raman scattering. Raman scattering is an inelastic scattering, which means that the frequency of the scattered light is different from the incident light. When the incident light, typically a laser, strikes on the target sample, most photons are scattered at the same energy level, leading to the same frequency. This is known as elastic scattering, called Rayleigh scattering [83]. However, there is also a small fraction of photons that are scattered at different frequency after interaction with the target samples and this is Raman scattering. The schematic of the Raman scattering process is shown in Figure 2d. In Raman scattering, the scattered photons have different energies, thus different frequencies and wavelengths compared to the incident photons. Raman spectroscopy utilizes Raman scattering for the identification at the molecular level due to the distinctive energy shift in scattering after interaction with different chemical bonds according to their vibrational modes [84]. The shifts caused by different chemical bonds are recorded as Raman spectra, and each bond corresponds to different peaks. According to the Raman spectra, the chemical structure of the molecule can be reconstructed [85].

Raman spectroscopy is explored for use in sensing applications. However, only a very small fraction of photons is scattered in this way and the Raman signal is usually very weak at low concentrations of the analyte. To improve the detection sensitivity, the emitted signal needs to be enhanced. Surface Enhanced Raman Spectroscopy (SERS) is such a surface technique that can be used to enhance the Raman signal by performing Raman measurements on plasmonic substrates such as metallic nanoparticles or nanostructures. 

Two primary mechanisms are responsible for SERS enhancement: electromagnetic and chemical enhancements, and electromagnetic enhancement is considered as the dominant one. When the incident light strikes the surface, Localized Surface Plasmons (LSPs) are excited. They will enhance the local electromagnetic field. The electromagnetic fields generated by Surface Plasmons (SPs) and LSPs at the surface of the metal will interact with the incoming photons and with the Raman emitted photons to provide significant enhancement of the Raman scattered photons. SERS allows for highly sensitive structural detection of low-concentration analytes through the amplification of electromagnetic fields generated by the excitation of LSPs [86].

To quantify the enhancement of the SERS signal due to the plasmonic substrate such as silver nanoparticles, the Enhancement Factor (EF) is a commonly used characteristic when designing a biosensor for SERS. There are different ways to define and calculate EFs, among which one of the widely used EFs is the Analytical Enhancement Factor (AEF), which is defined from an analytical chemistry point of view as: AEF = *-*$\frac{I_{SERS}/C_{SERS}}{I_{Raman}/C_{Raman}}$ [87], where $I_{SERS}$ and $I_{Raman}$ denote the measured intensity via SERS and Raman, respectively, while $C_{SERS}$ and $C_{Raman}$ are the concentration of analyte detected via SERS and Raman respectively. The EF parameter will ultimately play a role in the sensitivity of the device, with higher EF leading to better sensitivities and lower LOD.

Raman or SERS measurements can be achieved by a Raman system with different optical components. The incident laser is focused on the target samples using an optical lens. The focused laser then interacts with the target molecules, resulting in photons that are scattered at different frequencies that correspond to different chemical bonds. The scattered photons will pass through the dichroic mirror and finally reach the grating, where the scattered light at different frequencies (wavelengths) will be grated and recorded as a Raman spectrum by the detector such as a CCD detector. Based on the recorded spectrum, the target molecule can be identified.

One method for the direct identification of SARS-CoV-2 virus is to detect the surface proteins such as the spike protein. By measuring the chemical bonds from the protein, SERS can be used in this way to reconstruct and determine specific proteins. For example, Yang et al., designed gold nanoneedle arrays that are functionalized with ACE2 to trap SARS-CoV-2 virus [31]. They demonstrated the distinct Raman peaks of two viral strains that encode the spike protein and nucleocapsid protein of SARS-CoV-2 and established the identification standard using PCA to distinguish clinical positive samples with S and N proteins from negative samples. For serological tests in serum against COVID-19, SERS is reported to be combined with lateral flow immunoassay (LFA) for simultaneous detection of IgG and IgM to provide more information about the infectious stage [88]. 

In addition to noble metals such as gold and silver, other materials are also explored as SERS substrates for COVID-19 detection. Peng et al., for the first time, reported the development of a SERS biosensor using niobium carbide (Nb2C) and tantalum carbide (Ta2C) Mxenes and a limit of detection for the SARS-CoV-2 S protein reaching to the nM level [89]. 

Besides extensive experimental work related to the use of SERS biosensors for COVID-19 diagnosis, theoretical studies have been reported as well. As an example, Asma M. Elsharif designed a substrate by depositing SERS-active materials on PDMS inverted nanocavity arrays and simulated the enhancement factor distribution [90]. 

### 3.5. Interferometric Biosensors

Interferometry refers to the measurement of intensity changes when two coherent light beams are superimposed. The interference of two waves depends strongly on the phase difference. There are several basic types of interferometers: Michelson, Fabry–Pérot, Mach–Zehnder, Sagnac, and Young. Michelson interferometers consist of a beam splitter and two mirrors to achieve an interference pattern from two coherent lights. The light beam from a coherent light source, typically a laser, propagates to a beam splitter, where the light beam is partially reflected towards a mirror M1, and is partially transmitted through the splitter and propagating towards another mirror M2. Two split light beams are then reflected by each mirror and combined at the beam splitter with interference occurring. A detector such as a spectrometer or camera is placed on the other side of the beam splitter to record the interference pattern. It involves movable mirrors to adjust the optical pathlength. In a Fabry–Pérot interferometer, the optical cavity is mainly composed of two optical plates in parallel with partially reflecting surfaces facing each other. When the light is incident from a diffuse light source, partial light passes through the second plate, whereas partial light is reflected and bounces back and forth in between two surfaces. Thus, a series of light beams, after reflecting, pass through the second plate with a constant change in phase and interference with each other. For a conventional Fabry–Pérot interferometer, to detect the analyte with a biorecognition element, a reference measurement taken in an empty cavity is needed to identify the change caused by the binding of analyte. Mach–Zehnder interferometer, on the other hand, generates two light arms with a beam splitter. Each arm passes through a mirror, separately. These two arms then reach a second beam splitter, where each arm is split again and combined with the split beam from the other arm, generating two interference patterns that are recorded by two detectors. The Sagnac interferometer utilizes the interference of two split light beams that originate from the same light source but split and propagate in two opposite directions following same path. The two split light beams recombine and interfere with each other at the same point where they split (the coupling zone) [91]. The Young interferometer takes advantage of a double split to generate two light sources in coherence and then interference with each other. Depending on the incident angle, it is only when the difference of optical path is integer times of the wavelength that constructive interference occurs and maximum intensity is detected. Compared to Michelson and Fabry–Pérot interferometers, Mach–Zehnder and Young interferometers are more commonly used for the development of interferometric biosensors [92]. 

Interferometric biosensors measure the change in refractive index that is induced by the bioconjugate interaction (Figure 2h). When a light beam propagates in a medium with a refractive index n for a certain path length L, the phase change *ϕ* is determined by the following equation: *ϕ = 2 πnL/λ*, where *λ* is the wavelength [92]. However, utilizing an interferometer directly for biosensing isn’t ideal until a fixed surface is implemented for the immobilization of the bioreceptor. This is because the change in the refractive index of the sensing medium such as air or solution caused by the binding event of the analyte is very small and indistinguishable compared to the medium with no binding event. Therefore, to improve the sensitivity for an interferometric biosensor, an optical waveguide, of which the evanescent field is highly sensitive to the surface change, is integrated [93], and an interferometer is established on the surface of the waveguide. A waveguide is a physical structure that allows for the propagation and guidance of the electromagnetic wave. Based on the specific geometry, there are cylindrical and planar waveguides, with the planar waveguide being more commonly used in interferometric biosensors. When light is propagating and confined inside the waveguide core upon total internal reflection, an evanescent field exists closely at the surface of the waveguide due to boundary continuity. For the development of a biosensor, the surface of the waveguide can be modified with a recognition element (bioreceptor) that is specific for the target analyte. During the propagating of the electromagnetic wave, the binding of analyte molecules on the bioreceptor or the adsorption of the molecule to the surface causes the change in the effective refractive index (n_eff_) of the near-field environment in a cover medium with the interaction of the evanescent field and samples (Figure 2g). The change can be probed immediately by the evanescent field at the surface area, resulting in a phase change in the propagating wave [94], which can be expressed as *ϕ = 2 π*Δ*_neff_L/λ*. The calculation of Δ*n_eff_* is related to surface mass coverage/density upon molecular adsorption, and the relation is as expressed [95]: Δ*Γ* = $\frac{d\Gamma }{dt_{ad}}\frac{dt_{ad}}{dn_{eff}}$ Δ*n_eff_*, where $t_{ad}$ is the thickness of the adsorbed layer of the molecules, and its relation with surface mass density is described using De Feijter’s formula [96]: *Γ = t_ad_*$\;\frac{n_{ad}-n_{cover}}{dn_{ad}/\mathrm{dc}}$, where *n_ad_* and *n_cover_* are the refractive index of the adsorbed molecules and cover medium, respectively, and *c* is the concentration of the molecules.

Further, to extract/display the phase change of the sensing light that encounters analytes, a reference light beam that is split from the same light source is utilized. With the phase-changed wave, the interference signals can be obtained when detecting the reference light. Hence, information regarding the presence and quantity of the target analyte can be achieved. 

### 3.6. Ring Resonator Biosensors

An optical ring resonator configuration consists of a ring structure coupled with light input and output, which is typically realized by an optical waveguide. The waveguides for input and output are also known as bus or port waveguides [97]. Based on the number of the ring structured resonator, there are single-ring resonators, double-ring resonators, and multiple-ring resonators. In a simplest single-ring resonator with one ring waveguide (radius: r) and a port waveguide, the incident light from the input propagates within the port waveguide. When the light reaches the area that is close to the ring resonator, i.e., the coupling area, optical coupling via an evanescent field extending out of the surface of the waveguide occurs and allows some light to be coupled into the resonator. Since the amplitude of the evanescent wave decays exponentially along the distance, the distance between the linear waveguide must be very close to the ring resonator. Resonance occurs when the light inside the ring resonator constructively interferes with the light from the port waveguide. Therefore, the optical path difference, which is *2 πrn*, where *n* is the effective refractive index of the resonator, must be integer times the light wavelength. Following this requirement, the resonance wavelength is calculated to be: *λ_m_ = 2 πrn/m*, where *λ_m_* is the resonance wavelength, and *m* is an integer called the mode number [98]. In other words, only integer multiple normal modes of standing waves resulting from interference are allowed in this optical path length inside the resonator. For the input light with a wide range of wavelengths (e.g., white light), only the light with resonance wavelengths will be coupled with the ring resonator, and a decrease in transmitted intensity at these wavelengths will be observed in the output light as a function of wavelength. 

Some parameters are used to describe an optical ring resonator: Free Spectral Range (FSR), describing the distance of two neighboring resonance wavelengths, and Full-Width Half-Max (FWHM, i.e., bandwidth), describing the difference of two wavelengths with half the maximum intensity (when resonance occurs) within one resonance mode. Moreover, to characterize the resonator, Quality factor (*Q*) and Fineness (*F*) are used. *Q* is used to evaluate the free spectral range of a ring resonator and is expressed as $\frac{f}{FWHM}$, where *f* is the frequency and *FWHM* is the value for the transmission spectra. *F* is used to evaluate the narrowness of the resonance and is calculated as the ration of *FSR* to *FWHM*. 

Similarly to an SPR biosensor or interferometric biosensor, the ring resonator biosensor takes advantage of the fact that the refractive index is sensitive to molecular events such as a bioconjugate interaction. Therefore, the binding event of the analyte can be detected via the change in refractive index. In a ring resonator biosensor, a specific bioreceptor can be immobilized on the surface of the ring resonator within an evanescent field generated from the propagating wave in the resonator. In the presence of target analyte, the interaction of the analyte and the bioreceptor induces the change in the refractive index, further leading to the change in the resonance wavelength. With an optical detector, such as a spectrometer, the change in the resonance wavelength can be measured as a spectral shift and the analyte can be quantified based on the shift difference [99] (Figure 2i).

### 3.7. Photonic Crystal-Based Biosensors

A photonic crystal is composed of periodic nanostructures that interact with light in specific ways related to the periodicity of the structure and wavelength of the light [100]. This feature makes them attractive for biosensing applications. Photonic crystals can be fabricated in all three dimensions by generating periodic changes in the refractive index. For example, a one-dimensional photonic crystal can be fabricated by depositing multiple layers of thin films with different refractive indices; thus, the refractive index changes in a perpendicular direction along the deposited layers.

Considering a 1D photonic crystal structure that is composed of a low refractive index material with periodic nanostructures/gratings and a coated layer on the periodic gratings with a higher refractive index (a 1D slab surface), the photonic crystal structure can exhibit resonant reflection when interacting with incident light at a specific wavelength [101], which is caused by constructive interference between the reflected optical wave and a leaky waveguide mode, or guided resonance mode [102], resulting in a reflection peak in reflectance spectrum. The resonance wavelength *λ* is expressed as a function of the grating period *Λ* and the effective refractive index n: *λ = nΛ* [101]. 

Since the refractive index is affected by the surface properties, it is suitable for the development of biosensors. As shown in Figure 2j, the surface of the photonic crystal can be functionalized by immobilizing bioreceptor for a specific analyte. When the analyte of interest interacts with the bioreceptor on the surface, the refractive index is changed, thus the resonance wavelength is changed. A change in the resonance wavelength is caused, and a spectral shift in the reflectance spectrum will be observed, and the shift can be used to measure the concentration of the analyte.

### 3.8. Design Considerations for Photonic Biosensors

Different photonic detection techniques have been introduced with emphasis on their working principle, and parameters leading to quantitation were discussed. For the design of a photonic biosensor, it is complicated and difficult to provide a single, general equation, considering the requirement for different types of detectors and for different applications. For example, the optical readout configuration can be different for spectroscopy vs imaging devices. For colorimetric or fluorescence-based biosensors, where the intensity of photons is quantitatively measured via imaging such as a CMOS sensor, the sensing performance is determined by both the chemistry (such as the reaction efficiency, quantum yield of the luminophore/emitters in a chemiluminescence reaction) and the characteristics of the imaging system such as the numerical aperture and photon collection efficiency. The calculation of the recorded fluorescence intensity is related to these parameters, and the design should consider both the binding affinity and the detection system. As for plasmonic biosensors, such as SERS, the design is mainly focused on the substrate, whereas the EF is the parameter to be improved, as described in Section 3.4. For other photonic biosensors such as the SPR and interferometric biosensors that rely on a change in the refractive index of the sensing surface (induced by the binding event of the target analyte), the main parameter of the biosensor is the change in wavelength, resonance angle, and/or intensity that is caused by a change in the refractive index. The interactions between the analytes and bioreceptors are also critical factors that will affect the sensing performance. Therefore, the functionalization of the sensor that maximizes this interaction is another very important for the design of a biosensor.

The sensor performance, such as sensitivity and limit of detection, is measured via the calibration plot that describes the change of these parameters (wavelength shift, intensity, etc.) with analyte concentration (Figure 2IV). They are related to the binding event (analyte to the bioreceptor), on the one hand, and the transducing mechanism (such as refracting index, fluorescence, absorption, etc.) and the detection system, on the other hand. Taking a refractive index-based biosensor as an example, upon the binding event of the analyte to the bioreceptor on the functionalized surface, the refractive index on the sensing surface is affected, and this change is then detected in different ways (such as LSPR and SPR). As a result, in addition to the functionalization of the biosensor, the sensing performance of the biosensor is also closely related to the features/capabilities of the detector, such as its noise, quantum efficiency, and spectral response. The detector for some photonic biosensors can be the naked eye, which can distinguish, for example, differences between the test and control lines in colorimetric LFAs. However, the eye does not provide accurate quantitative results, and therefore, electronic detectors are used when quantitation is important. For luminescence, cell phone cameras that utilize a CMOS imaging sensor may be sufficient when the signal intensity is high, as demonstrated in our previous work [107,108]. The detection sensitivity relies on the capabilities of the CMOS sensor, such as quantum efficiency, spectral response, and noise. In addition, the optical configuration of the imaging sensor also affects the detection performance. Improvements in the configuration can lead to higher sensitivities. For example, in our previous work, the sensitivity for electrochemiluminescence detection is improved by adapting a single-electrode system with a microfluidic device that is directly attached to the surface of a CMOS sensor to achieve higher photon collection efficiency. To further improve the sensitivity, more sophisticated detectors are needed such as single photon counting devices that are able to record single binding events, leading to lower limits of detection. Moreover, the configuration of the instrument, such as the setup of optical components, will also affect the sensing performance. As an example, in one of our previous articles, we demonstrate an improvement in sensitivity for electrochemiluminescence detection by combining a single electrode system with a microfluidic platform that is prepared on the surface of a CMOS sensor to achieve higher photon collection efficiency [108].

### 3.9. Photonic Biosensors Applied to COVID-19 Diagnosis

As discussed in the beginning of this review article, for the diagnosis of COVID-19, biosensors can be designed to detect different analytes specific either to the virus itself, or to the immune response. Each technique described above exhibits advantages and limitations when used for different analytes. Although there are many biosensors on the market and ready for clinical diagnosis, most biosensors are still in an early stage and used for lab research or emergency only. To be implemented in practical applications or commercialized for COVID-19 detection, more characteristics need to be evaluated and validated to receive authorization. In this section, we will discuss the progress of different photonic techniques used in different types of testing and their advantages and challenges. In addition, the implementation for commercial products will also be discussed according to the standards or requirements from authorities.

Current gold-standard detection of COVID-19 is based on PCR. To visualize the result, the analyte can be labelled with fluorescent tags. There are also biosensors with different mechanisms developed for nucleic acid detection, such as the fluorescence-based toehold switch sensor for SARS-CoV-2 RNA detection [22]. The target viral RNA is amplified prior to detection. The toehold switch sensor is composed of a variable region with a toehold that is complementary with the target viral RNA, a ribosome binding site (RBS), and a translation start site (AUG) with the reporter gene LacZ, which can be easily detected by luminescence. In the presence of the trigger RNA, the toehold region interacts with it, leading to an alternate conformation, which enables the accessibility of RBS and AUG to the ribosome. The ribosome allows for the translation of the reporter gene that will be detected with a chromogenic substrate. 

Antigen tests and serological tests are more suitable for applications at the point of care or point of need. Compared to nucleic acid tests that usually require gene amplification, which further needs professional equipment and operators, antigen tests and serological tests make use of much simpler devices that can be easily accessed and used. Lateral flow immunoassay is a commonly known method that is developed for the detection of COVID-19. Many antigen tests and serological tests are realized by LFIA and/or ELISA via fluorescence, chemiluminescence, and colorimetric methods. LFIA performed on a cellulose-membrane-based portable platform is suitable for point-of-care and point-of-need applications. Moreover, it can provide rapid detection within 15 min, which is crucial for massive detection against a pandemic. ELISA, on the other hand, utilizes a micro-plate for detection of antigens or antibodies at larger quantity. Compared to LFA, it takes a longer time, typically ranging from 1 h to 5 h [109], but provides higher sensitivity and specificity [110]. 

Efforts have been devoted to the development of antigen and antibody tests via various optical methods, from laboratory research to commercial products. For example, Guo et al., proposed a fluorescence sensor combined with LFIA to detect the S and N proteins of SARS-CoV-2 by utilizing mesoporous silica-encapsulated up-conversion nanoparticles as a fluorescence reporter on LFA strips [24]. The reporter-labeled LFA strip is measured with a 5G-enabled portable device at the cm scale with an excitation laser and detector. This device can be connected with computers and smartphones via Bluetooth. A limit of detection as low as the ng/mL range is obtained. 

Plasmonics-based techniques such as SPR [29], LSPR [111], and SERS [112] are widely utilized for viral antigen detection. These methods provide high sensitivity and rapid detection with a response time ranging from a few minutes to half an hour. For the development of plasmonic biosensors for COVID-19 detection, the complex instrument and operation may be a challenge for point-of-need applications.

For the development of antibody tests via optical methods, in addition to the commonly used LFA and ELISA, chemiluminescence assays for serological tests have also been studied and developed as commercial products. For example, the Abbott AdviseDx SARS-CoV-2 IgG II assay that has been approved for EUA by the FDA can be used to help identify recent or prior infection with SARS-CoV-2 by detecting IgG antibodies in serum or plasma via chemiluminescence assay. The target IgG antibody will be linked to an anti-human IgG acridinium-labeled conjugate, which is the chemiluminescent molecule for detection. However, it can be used under emergency use authorization by laboratory professionals only, and the identification is semi-quantitative. Thus, it is not applicable for clinical use yet. The Roche Elecsys SARS-CoV-2 test is an electrochemiluminescence assay that utilizes a ruthenium complex (Tris(2,2′-bipyridyl)ruthenium(II)-complex (Ru(bpy))) as an electrochemiluminescent probe. It forms a sandwich structure with the target antibodies to SARS-CoV-2 and biotinylated SARS-CoV-2-specific recombinant antigen, which will be later linked to streptavidin-labeled magnetic micro-particles. However, these assays still require a specialized instrument for further analysis. Therefore, developing detection methods that are simple and easy-to-use is urgent and important.

## 4. Clinical Photonic Biosensors

### 4.1. Requirements for Clinical Use

To design an optical biosensor that can be used for clinical use, it is necessary to understand the requirements for approval by regulatory bodies. These requirements are related to the characteristics and limitations of their testing performance and may vary for different use cases. For example, both Health Canada and the FDA claim that serological tests are not appropriate for the diagnosis of COVID-19, but they can still be approved and used for identifying the prevalence of COVID-19 (https://www.fda.gov/medical-devices/coronavirus-disease-2019-covid-19-emergency-use-authorizations-medical-devices/eua-authorized-serology-test-performance, content updated on 3 December 2021, and accessed on 1 June 2022). Regarding the requirements for sensitivity and specificity for serological tests, different countries and regions have different standards. The table below (Table 3) shows a comparison of the target values for sensitivity and specificity required for devices used for serological tests.

Currently, there are different tests that have been approved for emergency use. A summary of the approved tests is shown as follows (Table 4):

### 4.2. Challenges for Clinical/Practical Use

The process of developing a commercial biosensor that can be approved or used in operational environments goes through several levels, each with its challenges. The challenges may vary depending on the type of technology and the requirements for its application. For a biosensor used for the diagnosis of COVID-19, considering the imperative need for massive rapid tests, it is important to develop a biosensor that provides a method that is accurate and rapid, as well as simple for operation, so that no complicated equipment and professional operation, which require extra training and time for personnel, are needed. Consequently, the challenges come from the high standards for fast response time, simplicity of operation, and low cost for the device and reagents. 

**Figure 3:**
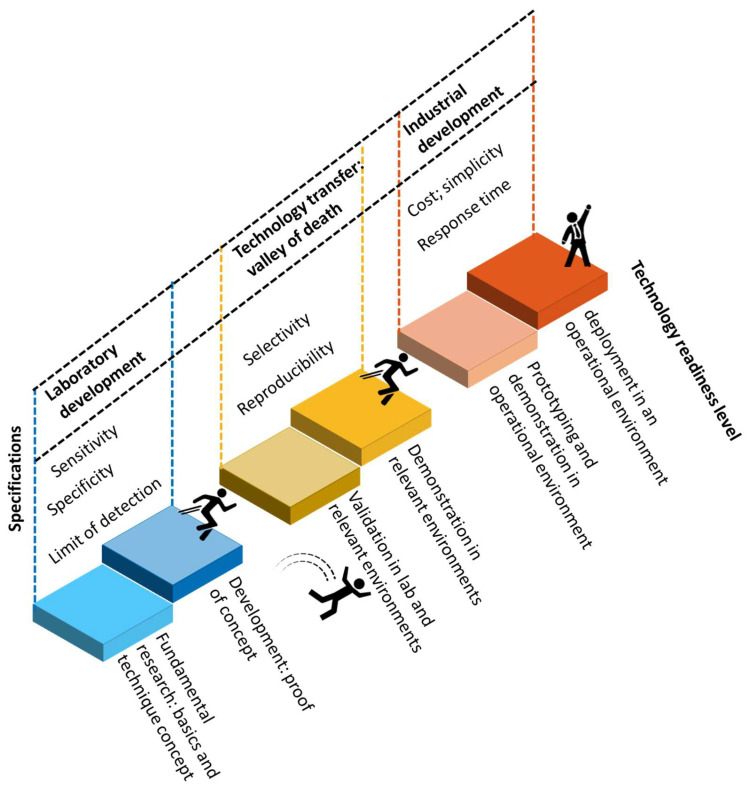
Challenges during the different development stages of a biosensor for commercialization/ready-to-use.

In the fundamental research stage (Figure 3: Laboratory development), researchers are focused on the development of a proof of concept of a biosensor or a sensing device. To achieve this, a biosensor is firstly designed with specifications according to the intended application. Prior to the design specifications, each component of a basic biosensor should be selected or decided, including analyte preparation, bioreceptor (typically achieved by functionalization) and detecting method or technique (for the transducer). For biosensors that are designed for COVID-19 testing, the analytes can be, as described earlier, viral RNA, membrane protein, or immunological antibodies (IgG and/or IgM), based on the type of testing. They can also be designed for multiplexing to achieve higher accuracy. For example, many biosensors that are used for serological tests can detect both IgG and IgM antibodies [27]. For a biosensor in this stage, the technical characteristics as discussed before are the most important parameters, among which the potential applications are significantly dependent on the sensitivity, LOD, and specificity. Since there are different types of tests for COVID-19 detection using a biosensor and the analytes detected can be different, there is currently no normalized/standard sensitivity or LOD for the development of a biosensor. 

After the development of a biosensor with high sensitivity and specificity and an acceptable limit of detection (LOD) in the laboratory, it is important to validate and demonstrate that its performance reaches the relevant criteria prior to transferring the developed biosensor to practical applications. In this stage (Figure 3: Technology transfer), clinically relevant data are collected, and the performance of the new biosensor device is compared with the gold standard to build the confusion matrix and calculate the clinical parameters/characteristics of the biosensor (see Section 2.2). If a gold standard does not exist, reference against other clinical data can be made, or validation without a gold standard can be pursued. In the case of COVID-19, the gold standard is a PCR test. This is the stage when many technologies may fail and the trip towards industrial implementation or commercialization ends. The biosensors developed in the lab should be able to quantitatively detect specific genes from viral RNA or proteins with the required sensitivity, LOD, and specificity. The developed biosensors will be further validated in the lab with simulated real samples and/or in relevant environments. Herein, sample collection and preparation are critical for the development of a biosensor in this stage. For nucleic acid and antigen tests, samples are usually collected by nasopharyngeal swab. For antibody tests, most measurements are conducted in blood samples. More substances in the sample will be introduced and they may interfere the detection accuracy. Therefore, in this stage, the challenges are usually related to selectivity and specificity while maintaining high sensitivity. Interferences from other substances such as coronavirus can affect the clinical performance, leading to false positive results when using a biosensor with poor selectivity and specificity. This is particularly challenging for the validation in a relevant environment. Therefore, it is important to overcome or address these challenges from the beginning of the fundamental research to avoid further difficulties in the transferring stage.

After the success of the validation and demonstration, the developed biosensor shows high potential for application in real environments. This is when other parameters such as cost, response time, and simplicity of operation attract more attention, since the biosensor will be delivered soon to end-users. The developed biosensors will be prototyped and authorization from government will be needed to be deployed in operational environments (Figure 3: Industrial development). Based on different criteria from different countries or regions (Table 3), evaluation of performance for approval is usually based on clinical characteristics, which are more straightforward parameters such as PPV and NPV. To evaluate the clinical characteristics in operational environments, measurements with nasopharyngeal swab specimens that are collected from patients with symptoms are typically performed and the results are compared with the standard method for the calculation of these parameters. To be approved and implemented in practice, the results must meet the required standards provided by relevant institution. 

### 4.3. Current Status of Photonic Biosensors in Commercialization

**Figure 4:**
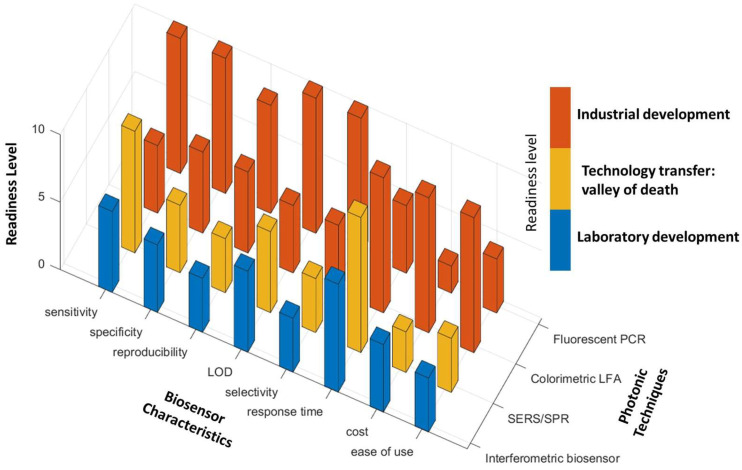
Prospects of different optical techniques for COVID-19 testing. The x coordinate indicates difference biosensor characteristics, the *y* axis indicates techniques, and the z coordinate represents the relative performance of each technique for its characteristic feature. The color of the column refers to the technical readiness level of each technique indicated in arbitrary values. The higher the value of z coordinate is, the more mature and advantageous the characteristic of a specific technique is, compared to other techniques. It is worth noting that this figure shows a relative comparison of different techniques’ characteristics and features in general. When it comes to a specific product or device, the characteristics may vary depending on how it is designed and developed. It is possible that a specific product based on a high-readiness-level technique is not ready on market yet due to other factors.

As discussed above, many factors play important roles in the development of a biosensor for COVID-19 diagnosis. For each photonic technique, there are different advantages and disadvantages that affect its current status or readiness level (as shown in Figure 4). To demonstrate a straightforward comparison between different techniques in different aspects, a scale of values from 0 to 10 is used to represent the readiness level. These numbers are our own estimates based on the literature reported and the availability of different techniques on the market.

Colorimetric biosensors are commonly used in serological tests (LFA and ELISA). Sample extraction and amplification play key roles for the sensitivity and specificity. During the validation stage in real environments, this might be challenging. LFA and ELISA are typically used for a serological test and are used for the detection of IgG and/or IgM. These methods may suffer from poor sensitivity. Therefore, it is more important and necessary to improve the sensitivity and specificity for LFA- and ELISA-based biosensors. As mentioned above, there are various approaches for each colorimetric technique to increase signal, hence improving the sensitivity. 

Fluorescence-based detection is utilized widely in nucleic acid tests such as fluorescence-based PCR, which provides high specificity and sensitivity. Fluorescence-based LFAs and ELISAs have also been explored and applied in practice widely due to their advantages such as high sensitivity, ease of use, and rapid response. Numerous biosensors based on these techniques have been developed and commercialized and have already laid the foundation for future progress. However, on the other hand, it also makes the barrier of entry for new technologies to the market more competitive. For researchers, to further develop biosensors in this field, addressing existing problems such as the complex operation of fluorescence-based nucleic acid tests and their high cost will be critical and necessary.

Unlike the above-mentioned methods, most plasmonic and refractive-index-based biosensors are still limited to lab research use only. Refractive-index-based photonic techniques such as SPR usually utilize the change in refractive index due to the binding interaction between antigen (SARS-CoV-2) and antibody (IgG, IgM), or the hybridization of viral RNA with a complementary nucleic acid, allowing for highly sensitive detection. Asghari et al., discussed the calculation of the effective refractive index of the virus SARS-CoV-2 [118]. Plasmonic biosensors such as SERS can also provide accurate detection of COVID-19 by detecting the characteristic bands corresponding to the virus or antibodies without labels. However, considering the complex composition of the analytes, the label-free method may suffer from poor specificity. Probe molecules such as 4ATP can address this issue and are commonly used for SERS detection to provide a strong signal [119]. Labeled detection, on the other hand, may improve the complexity. Therefore, for the development of a biosensor, the balance between technique and the performance is important. Depending on the desired specification, researchers can design the approach accordingly. 

In summary, biosensors based on colorimetry and fluorescence, especially LFA, ELISA, and PCR, are more mature technologies on the market, whereas plasmonic biosensors such as SPR and SERS are under rapid development to take them out of the lab. The specific use case determines the features and characteristics of each device. For example, LFA may suffer from poor sensitivity, but it is still commonly utilized due to its ease of use and low cost. These advantages make LFA a great candidate for rapid detection, particularly for massive measurements that are urgently needed for the pandemic. On the other hand, although PCR takes a longer time and professional operation, it still is the gold standard benefiting from its high sensitivity and specificity. For plasmonic and refractive-index-based biosensors, they have the potential to provide balancing features for ease of use and sensitivity, but more efforts need to be dedicated towards taking them to the market.

## 5. Conclusions and Perspectives

In conclusion, we reviewed current photonic techniques that have been explored for the diagnosis of COVID-19, and discussed their advantages, limitations, and challenges, as well as the current status of their technical readiness level to be used for clinical applications. To date (July 2022), more than 500 million infected cases have been reported. The large number of infected cases indicate that large-scale testing is needed, which is associated with a high economic burden. Moreover, as we now know, asymptomatic individuals can also spread the virus, resulting in the increased importance and added challenges of limiting the transmission of the virus. So far, according to the statistics conducted by Our World in Data, more than 11 billion tests (laboratory tests including both PCR tests and/or antigen tests) have been performed globally, and the number of total tests performed is continuously increasing. Among these tests, the most tests are performed in China (~9 billion nucleic acid tests), the United States (~900 million PCR tests), India (~850 million PCR and antigen tests), and Italy (~200 million PCR and antigen tests). On average, more than 10 billion PCR tests have been performed since WHO declared COVID-19 a pandemic until July 2022, accounting for most of the total costs incurred. Assuming the cost of a PCR test for SARS-CoV-2 is similar to SARS-CoV, a virus from the same family, the cost per PCR test ranges from approximately $10 to $50 [120]. Considering the average cost of a PCR test to be $30, 10 billion tests will cost approximately $300 billion, with an average rate of daily spending of approximately $0.35 billion. If a less expensive test kit, such as a home test kit that costs $10, is developed, it could save about $0.23 billion per day that is currently spent on mass testing. Therefore, investment in developing testing technologies with high sensitivity and specificity but lower cost would have a significant overall impact on the health care system, as funds can be redirected towards the development of treatments and improvements in infrastructure. The development of low-cost testing technologies can not only ease the financial burden incurred by testing alone but can also have an impact on the disease burden (DALYs), as well as reduce the economic loss caused by DALYs. Specifically, testing and diagnosis of COVID-19 can help people to identify the disease and receive timely isolation and treatment, thus limiting the spread of virus and reducing the severity of the disease as well as the risk of death or long-term disability. Consequently, the economic loss due to DALYs will be reduced. The economic loss can be estimated by multiplying DALY lost due to COVID-19 and Goss Domestic Product (GDP) per capita [121]. Taking Europe as an example, as mentioned in Introduction, the DALY loss of 16 European countries is approximately 4000 years per 100,000 population in 2020, and the GDP loss per capita of Europe in 2020 was approximately US $34,000. Hence, the economic loss of every 100,000 people is $136 million. Based on a study that simulates the cost-effectiveness of testing strategies for Wuhan (with a total population of approximately 8,000,000), conducting three times the PCR tests versus two times increased the Quality-Adjusted Life Years (QALYs) by 850.1 years, with 749.4 QALYs due to the averted YLLs and 100.7 QALYs due to the increased Quality-Adjusted Life Days (QALDs) representing fewer sick days [122]. Assuming all population in Wuhan received two PCR tests at a price of $30, the total cost of testing is $480 million. For a three-test strategy and a lower-cost device of $10, the cost of testing is $240 million (saving $240 million), with additional gains from the monetary value of the averted YLLs (~750 QALYs × $18,368 GDP/capita of Wuhan = $13.7 million), leading to a total net gain of $253.7 million.

These examples illustrate the need for taking testing devices out of centralized laboratories and for developing cost-effective biosensors that can be easily accessed and used at the point of need. Currently, different countries and regions are promoting the development of such diagnostic testing devices. For example, Canada is fostering the development of testing devices by providing support for research and development from discovery to the market, as well as nurturing collaborative efforts between different entities each focused on engineering, medical relevance, and social impact. So far, more than 100 testing devices for COVID-19 have been approved in Canada, and more testing devices providing more options for use cases (e.g., self-test, point-of-care) and/or technologies such as multiplexing and breathalyzers are still under development and expected to be approved and distributed to end users across the country. This opens significant opportunities for both researchers and entrepreneurs to develop novel techniques and products for COVID-19 testing.

With increasing effort for the development of photonic biosensors for COVID-19, more and more technologies are now ready for the transition from the laboratory to the market. Photonic biosensors such as fluorescent and colorimetric biosensors have been widely used for rapid detection of SARS-CoV-2 due to their simplicity and ease of use on a portable platform, such as LFA. In comparison, biosensors based on other photonic techniques such as SERS and SPR are still in an early stage of readiness level for real environmental application and commercialization. These techniques are advantageous for their high sensitivity and specificity, as well as the rapid response. However, although they are widely used, there are still limitations existing in these techniques. For example, colorimetric biosensors suffer from poor sensitivity and specificity, which make them less reliable. The other photonic biosensors with high sensitivity require professional equipment, which limits their application in decentralized environments. Hence, for the development of photonic biosensors in the future, it is important to address the drawbacks of current techniques. 

One strategy is to combine different approaches. One example is to combine colorimetric biosensors with other highly sensitive techniques. For example, LFA can be combined with photonic detection such as by SERS by labelling the target analyte with a SERS active probe. In this way, the quantification can be significantly improved, thus addressing the problem of a colorimetric sensor that has poor accuracy. Another way is to explore new technologies to address the limitations of a specific technique. For instance, the PCR test is expensive, time-consuming, and requires professional equipment; other techniques such as LAMP can be deployed to perform RNA amplification for nucleic acid tests with high sensitivity. Compared to PCR, LAMP doesn’t require thermal cycling and can generate results within 30 min. As a result, it is more cost-effective and rapid. In addition to technology improvements, there are other strategies that can be explored to improve the accuracy of testing. For example, for antigen tests, a serial test can be implemented to measure the changing antigen levels. Two to three antigen tests can be conducted over a certain period of time. In this way, it can be used to screen asymptomatic people. Another example of a testing strategy is group testing that can reduce the cost when samples from multiple individuals (usually up to 10) are mixed as one sample and detected via a single test [123]. Group testing improves the testing efficiency significantly (up to 10-fold), and it can used to screen the potentially infected groups. Once a group test is positive, further single tests for people in this group are needed to confirm the infected individual. 

COVID-19 has been a part of our life and its impact will last for a long time even after the pandemic is over. With increasing attention being paid to the importance of testing for COVID-19, the development of testing devices for COVID-19 can progress faster. Researchers worldwide are devoting their efforts towards the development of accurate, rapid, and inexpensive testing technologies that can make a positive impact on the current situation. As discussed throughout the article, an improved test can reduce the economic and social burden and therefore it is paramount to fast track available and newly developed technologies through the process of commercialization. It requires, however, significant financial support for all stages, from ideation to commercialization. We strongly believe that, due to their availability, potential for low cost, and ease of use, optical technologies will have a particular role in this effort.

## Figures and Tables

**Table 1:** Clinical characteristics of a biosensor.

Total Population	Patient (Infected)	Heathy (Not Infected)	PPV and NPV
Test positive	True Positive (TP)	False Positive (FP)	PPV = $\frac{TP}{TP+FP}$
Test negative	False Negative (FN)	True Negative (TN)	NPV = $\frac{TN}{TN+FN}$
Sensitivity and specificity	TPR = $\frac{TP}{TP+FN}$	TNR = $\frac{TN}{TN+FP}$	-

**Table 2:** Photonic biosensors for COVID-19 diagnosis.

Method	Analyte	Mechanism	Sensitivity/ LOD	Response Time	Limitation	Ref.
Chemiluminescence	Anti-S protein antibody and anti-N protein antibody	Nanoluciferase enzymatic luminescence reaction active by binding of SARS-CoV-2 antibody.	89% for anti-S protein antibodies and 98% for anti-N protein antibodies	30 min	Lacking further evaluation and validation	[21]
	IgG antibodies	IgG binds to anti-human IgG acridinium-labeled conjugate, which is the chemiluminescent molecule for detection.			Semi-quantitative, laboratory level	Abbott AdviseDx SARS-CoV-2 IgG II assay
Luminescence	SARS-CoV-2 RNA	In the presence of the trigger RNA, the toehold region interacts with it, leading to an alternate conformation, which enables the accessibility of RBS and AUG to the ribosome. The ribosome allows for the translation of the reporter gene that will be detected with a chromogenic substrate.	100 copies of SARS-CoV-2 RNA	30 min	Target viral RNA is amplified prior to detection	[22]
Luminescence-based LFIA	SARS-CoV-2 S and N protein antigen	A magnetic quantum dot with a triple quantum dot shell (MagTQD) as a nanotag.	0.001 ng/mL			[23]
Fluorescence	S and N proteins of SARS-CoV-2	Utilizes mesoporous silica encapsulated up-conversion nanoparticles as a fluorescence reporter on LFA strips.	ng/ml			[24]
LFA/colorimetric	IgM antibody	Colloidal AuNPs as a reporter.	100% sensitivity	15 min	Quantitative is still problematic	[25]
	anti-SARV-CoV-2 IgG	Utilizes lanthanide-doped polysterene nanoparticles.		10 min		[26]
	IgG and IgM	Selenium nanoparticle.	94.74%	5 min		[27]
LFA	IgG and IgM	Superparamagnetic nanoparticles (SMNPs) and a giant magnetoresistance (GMR) sensing system.	10 ng/mL for IgM and 5 ng/mL for IgG	10 min	More complicated compared to conventional LFA	[28]
SPR	SARS-CoV-2 S protein	Virus is sandwiched within AuNRs-SARS-CoV-2 spike protein antibody and a Au-nanosheet-coated prism.	111.11 deg RIU^−^^1^	A few min	Mostly simulation	[29]
LSPR	Anti-SARS-CoV-2 S protein antibody	Au nanospikes on a glass.	∼0.08 ng/mL (∼0.5 pM)	30 min		[30]
SERS	SARS-CoV-2 S protein	Gold nanoneedle arrays that are functionalized with ACE2 to trap SARS-CoV-2 virus.	~7.88 ng/mL (17.7 pM)	5 min		[31]
	S protein	A colloidal AgNP-based SERS aptasensor utilizing the aptamer RBD-1C	5.5 *×* 10^4^ TCID_50_/mL	7 min		[32]
SERS-LFA	IgG and IgM	target sample is conjugated with SiO_2_@AgNPs that are modified with Raman test probe DTNB.				[33]

**Table 3:** Required sensitivity and specificity for serological tests. (Table source: Health Canada, https://www.canada.ca/en/health-canada/services/drugs-health-products/covid19-industry/medical-devices/testing/serological/notice-sensitivity-specificity-values.html, content published on 24 June 2020, and accessed 1 June 2022).

Country	Sensitivity	Specificity
UK MHRA	>98% (95% CI 96%-100%) on specimens collected 20 days or more after the first appearance of symptoms	>98% (95% CI 96%-100%)
US FDA	**Serology test:** 90% overall 70% IgM 90% IgG	**Serology test:** 95% (overall-total)
	**Nucleic acid:** >95% (lower bound of the 2-sided 95% confidence interval > 76%)	**Nucleic acid test:** ≥98% (with a lower bound of the 2-sided 95% confidence interval > 95%)
Health Canada	95% for IgG or total antibodies in samples collected 2 weeks or more after symptom onset	98%

**Table 4:** EUA-granted tests for at-home use.

	Sample	Analyte	Mechanism	Transducer	Time to Results	PPV/NPV (%)	LoD	Reference
**Molecular Tests**								
Lucira All-In-One COVID-19 Test Kit	Self-collected nasal swab	SARS-CoV-2 RNA of N gene	RT-LAMP w/a pH-mediated colour change of halochromic agents	Optoelectronic	<30 min	94.1%/ 98.0%	900 cp/mL of VTM (2700 cp/swab)	[113]
Lucira CHECK-IT COVID-19 Test Kit	Self-collected nasal swab	SARS-CoV-2 RNA of N gene	RT-LAMP w/a pH-mediated colour change of halochromic agents	Optoelectronic	<30 min	94.1%/ 98.0%	900 cp/mL of VTM (2700 cp/swab)	[114]
Cue COVID-19 Test	Anterior nasal swab	SARS-CoV-2 RNA of N gene	Isothermal nucleic acid amplification	Electrochemical	20 min	92.0%/ 98.0%	1300 cp/mL of sample	[115]
**Antigen Tests**								
BinaxNOW COVID-19 Ag Card Home Test	Anterior nasal swab	SARS-CoV-2 nucleocapsid protein antigen	Colorimetric lateral flow immunoassay	Optical	15 min	91.7%/ 100.0%	140.6 TCID_50_/mL	[116]
BinaxNOW COVID-19 Antigen Self-Test	Anterior nasal swab	SARS-CoV-2 nucleocapsid protein antigen	Colorimetric lateral flow immunoassay	Optical	15 min	91.7%/ 100.0%	140.6 TCID_50_/mL	[117]

## Data Availability

Not applicable.
